# Biofilm Surface Density Determines Biocide Effectiveness

**DOI:** 10.3389/fmicb.2017.02443

**Published:** 2017-12-08

**Authors:** Sara Bas, Mateja Kramer, David Stopar

**Affiliations:** ^1^Department of Food Science and Technology, Biotechnical Faculty, University of Ljubljana, Ljubljana, Slovenia; ^2^Lek d.d., Sandoz, Ljubljana, Slovenia

**Keywords:** biofilms, *E. coli*, biocide, antimicrobial, surface coverage, viscoelasticity

## Abstract

High resistance of biofilms for chemical challenges is a serious industrial and medical problem. In this work a gradient of surface covered with biofilm has been produced and correlated to the effectiveness of different commercially available oxidative biocides. The results for thin *Escherichia coli* biofilms grown in rich media supplemented with glucose or lactose on glass or poly methyl methacrylate surfaces indicate that the effectiveness of hydrogen peroxide or chlorine dioxide and quaternary ammonium compounds is inversely proportional to the fraction of the surface covered with the biofilm. In areas where biofilm covered more than 90% of the available surface the biocide treatment was inefficient after 60 min of incubation. The combined effect of oxidant and surfactant increased the effectiveness of the biocide. On the other hand, the increased biofilm viscoelasticity reduced biocide effectiveness. The results emphasize differential biocide effectiveness depending on the fraction of the attached bacterial cells. The results suggest that biofilm biocide resistance is an acquired property that increases with biofilm maturation. The more dense sessile structures present lower log reductions compared to less dense ones.

## Introduction

High resistance of biofilms for chemical challenges is a serious industrial and medical problem (Parsek and Fuqua, 2004). There are multiple mechanisms of bacterial resistance which vary with the bacteria present in the biofilm and the drug or biocide being applied (Videla, 2002). These mechanisms include physical or chemical reaction–diffusion barriers to antimicrobial penetration into the biofilm, slow growth of the biofilm cells due to nutrient limitation, activation of the general stress response, and the emergence of a biofilm-specific phenotype (Mah and O’Toole, 2001; Stewart, 2002). The individual bacteria in a biofilm may undergo physiological changes that improve resistance to biocides, such as induction of the general stress response (e.g., *rpoS*-dependent process in Gram-negative bacteria), increased expression of multiple drug resistance pumps, activation of quorum-sensing systems, or changing profiles of outer membrane proteins (Mah and O’Toole, 2001). Furthermore, biofilms are rarely, as the name implies, continuous films of microbial material that cover large surface area but are uneven distributions of small and large patches of biofilm structures. It is generally assumed that biocide effectiveness in biofilms is approximately three orders of magnitude lower compared to bacterial suspensions (Mah and O’Toole, 2001). However, this might be misleading as biofilms are inherently heterogeneous structures and different parts of the biofilm may have significantly different susceptibilities for biocides. The effect of surface coverage heterogeneity in biofilms on biocide effectiveness has not been systematically studied yet.

Biofilm heterogeneity spans different spatial scales (Karimi et al., 2015). Ultimately the effectiveness of the biocide will depend on the non-homogeneous distribution of individually attached cells. Antimicrobial agent must gain access to the heterogeneous biofilm structure. The rate of diffusion across a biofilm surface is dependent on the temperature, molecular size, and concentration gradient of the diffusing molecule (Watanabe et al., 1999; De Kee et al., 2005). Another important parameter is surface to volume ratio. The increased surface to volume ratio decreases time for the antimicrobial to diffuse through the volume. Under several environmental conditions biofilms may form thin flat structures with high surface to volume ratio, where vertical dimension of the biofilm is much smaller than the horizontal dimensions (Heydorn et al., 2000; Wimpenny et al., 2000; Young, 2006; Liu et al., 2015). In such cases the biofilm can be considered as a thin slab-like surface through which the antimicrobial agent diffuses. The two factors that are particular to diffusion through a slab-like structure are surface area and permeability, the latter being dependent on viscoelasticity (Peterson et al., 2015). It is hypothesized that in a thin biofilm structures the effectiveness of a biocide can be directly related to the surface area covered by the biofilm.

Because of their broad-spectrum activity against a variety of organic compounds oxidative biocides are often used in industry in control of biofilms (McDonnell and Russell, 1999). Oxidative biocides are proposed to have multiple targets within a cell which include peroxidation and disruption of membrane layers, oxidation of thiol groups, enzyme inhibition, oxidation of nucleosides, impaired energy production, disruption of protein synthesis and, ultimately, cell death (Finnegan et al., 2010). Often used biocides such as H_2_O_2_ and ClO_2_ that involve free radical formation can change amino acids, peptides, and proteins through hydrogen abstraction, electron transfer (oxidation or reduction), addition, fragmentation and rearrangement, dimerization, disproportionation, and substitution (concerted addition and elimination) (Finnegan et al., 2010). H_2_O_2_, *per se*, is considered a weak oxidant agent. However, it can easily cross the cellular membrane and reacts with transition metals, generating a highly reactive OH⋅ that may oxidize and fragment the protein or DNA backbone (Hawkins and Davies, 2001). On the other hand, the primary mechanism for inactivation of the *Escherichia coli* with ClO_2_ is disruption of the protein synthesis pathway by inhibition of enzymes or interference with nucleic acid–amino acid complexes (Roller et al., 1980; Lin et al., 2016). Surfactants may be added to oxidizing agents to improve their antimicrobial effectiveness (Nakata et al., 2010). Among them, cationic surfactants such as quaternary ammonium compounds (e.g., CTAB) are frequently utilized for disinfection and sanitation purposes in a variety of fields, such as hospitals, food manufacturing, and pharmaceutical industry. Cationic surfactants disrupt cell membrane, inhibits the activity of Mn-SOD and SoxS, cause leakage of intracellular K^+^ and other cell components, induce cell autolysis, and inhibit respiration (Nakata et al., 2010).

In this work, the impact of surface coverage on the biocide effectiveness was studied. To experimentally check this, a biofilm surface coverage gradient was produced and the effectiveness of different biocides was studied in glucose/glass and lactose/poly methyl methacrylate (PMMA) thin *E. coli* biofilms. Commercially available Klercide B and Klercide C biocides as well as lab made solution of 6% H_2_O_2_ were used as biocides. A gradient in biofilm surface coverage has been created by growing biofilms in a Falcon tube with silica glass or PMMA slides positioned in a vertical direction in rich media supplemented with glucose or lactose under slow mixing conditions. A gradient of surface covered with biofilm formed from the air–water interphase to the bottom of the tube. The surface covered with biofilm was arbitrarily divided into three regions of high, medium, and low surface coverage. The antibacterial effectiveness of different oxidative biocides was tested. The results suggest a significant variability in antimicrobial effectiveness ranging from high effectiveness in area with low surface coverage to ineffective antimicrobial treatment in areas with high biofilm surface coverage. The biocide effectiveness was reduced with increased biofilm viscoelasticity.

## Materials and Methods

### Bacterial Strain and Media Preparation

*Escherichia coli* MG1655, with plasmid gfp marker and resistance to kanamycin was grown overnight in LB Broth (Lennox, Laboratorios Conda) at 200 rpm, 37°C for 16 h. Two percent (v/v) of the overnight culture was transferred to fresh growth medium and grown to the mid of the exponential phase (OD_600_ 0.5) and transferred to biofilm reactor. Biofilms were grown in the rich growth medium with 1.88 g/L KH_2_PO_4_, 2.6 g/L Na_2_HPO_4_, 10.0 g/L peptocomplex, and 5.0 g/L yeast extract supplemented with either 22.0 g/L of glucose for glass surface biofilms or 23.1 g/L of lactose monohydrate for PMMA surface biofilms (Gomes, 2011).

### Biofilm Growth

A simple batch culture biofilm reactor was used for biofilm growth (Król et al., 2011). Sterile microscope slides 25 × 75 mm (glass or PMMA) were submerged in 25 mL of growth medium in a 50-mL conical tube and incubated at 37°C on an orbital shaker at 50 rpm. To grow the biofilm, the tubes were inoculated with 50 μL of the bacterial culture. The slides were transferred daily to tubes with a fresh growth medium. The loosely attached cells were not removed by rinsing before the transfer of each slide to the new medium. Biofilms were grown for 24, 48, and 72 h. Prior to the inoculation the glass and PMMA slides were sonicated in a water bath sonicator (ASonic Pro Med 50) for 5 min with maximum power in 70% (v/v) ethanol to remove impurities from the surface. Next the slides were treated with 6% (v/v) H_2_O_2_ in water bath sonicator for 10 min with maximum power and rinsed with Milli-Q water (Ahmed and Russel, 1975). The nonattached cells were rinsed with 3 mL of PBS by pipette.

### Biofilm Microscopy

Biofilms were observed after 24, 48, and 72 h under differential interference contrast (DIC) and brightfield microscopy. Slides were examined with Axio Observer Z1 epifluorescence microscope (Zeiss, Göttingen, Germany). The DIC and bright field images were observed using 10× and 20×, NA 1.4, Zeiss lenses. Images were recorded with a coupled MRm Axiocam camera (Zeiss, Göttingen, Germany). To determine the fraction of the surface that was covered with biofilm, the glass and PMMA slides were air-dried, biofilm was flame fixed, stained with Gram’s crystal violet solution (Sigma–Aldrich) for 15 min, rinsed with distilled water, and air-dried prior to the observation. For each incubation time the slides with biofilms were divided vertically into 25 slabs, each 0.8 mm wide. The first slab was positioned at the biofilm water–air interphase. Brightfield images were recorded at a center of a given slab at 10× magnification and were analyzed with ImageJ to determine the fraction of the surface covered with the biofilm. Gray-scale intensity threshold was set automatically and then manually adjusted to exclude the background. As the biofilm surface coverage decreased from the interphase to the bottom of the tube each slab was arbitrarily classified into high, medium, or low covered biofilm surface. Micrographs which had between 90 and 100% surface covered with biofilm were classified as high-density Zone I biofilms. In Zone II, biofilms from 10 to 90% of the available surface was covered with biofilm, in Zone III, less than 10% of the available surface was covered with biofilm structures. The length of the zone was determined with program AxioVision (Zeiss, Göttingen, Germany) with a length function. Next, the average fraction of surface covered with biofilm in a given zone was calculated. The total slide surface covered with biofilm (mm^2^) in a given zone was calculated by

(1) $$\begin{array}{c} A\;=\;f\;\cdot \;l\;\cdot \;b, \end{array}$$

where *f* is the average fraction of surface covered with biofilm in a given zone, *l* is the length of the zone, and *b* is the width of the microscopic slide. DIC microscopy was used to determine thickness of the biofilm structures. Cells at the top of the biofilm have been focused next the object slide was moved to the clean spot and the total height of the biofilm was estimated. The height in the most dense Zone I did not exceed five cell layers. On average, the biofilms in Zone I were 2–3 layers thick. Thickness in Zones II and III was lower.

### Antimicrobial Treatment

Biofilms grown for 24, 48, or 72 h were treated with 6% peroxide solution (200 mL of 30% H_2_O_2_ mixed with 800 mL PBS), Klercide-CR Filtered Biocide B (Shield Medicare, Ecolab), and Premier Klercide-CR Sterile Filtered Biocide C (Shield Medicare, Ecolab). According to the manufacturer Klercide-CR Filtered Biocide B (abbreviated Klercide B in this study) is a sterile cleanroom biocide that consists of a blend of stabilized chlorine dioxide and a quaternary ammonium compound. It has a broad spectrum activity and possesses fast kill rates even under conditions of heavy organic soiling such as high covered surfaces with biofilms. Premier Klercide-CR Sterile Filtered Biocide C (abbreviated Klercide C in this study) is a blend of 6% H_2_O_2_ and deionized water. For antimicrobial treatment the biofilms on glass or PMMA slides were rinsed with sterile PBS (3 mL of PBS applied with pipette) and inserted in Falcon tubes with 30 mL of antimicrobial agent or 30 mL of PBS as a negative control. Biofilms were treated for 2, 20, and 60 min at room temperature. Next, slides were rinsed with 3 mL of PBS. BacLight Bacterial Viability Kit (SYTO 9/propidium iodide) was used for live/dead stain (Invitrogen, 2009). Cells with intact membrane emit green light due to SYTO 9 (Em. 480/Ex. 500), on the other hand cells with compromised membrane (dead cells) emit red light due to propidium iodide (Em. 536/Ex. 617). To each slide 20 μL of stain mixture (1 mM SYTO and 6 mM propidium iodide in PBS) was added and covered with opaque cover glass (24 × 60 mm). The samples were stained for 30 min and observed with epifluorescence microscope (Zeiss, Göttingen, Germany) with appropriate settings for fluorescence (filters 38HE and 43HE).

Images were taken at 20× magnification. At least six randomly selected view fields per individual biofilm zone were examined and analyzed with ImageJ to determine the number of live and dead cells. The threshold was set to discriminate bacterial fluorescence intensity from the background intensity. Next, the total fluorescence intensity for a view field was determined and the number of viable bacteria was calculated by dividing the total green fluorescence intensity with the intensity of the single bacterial cell. In total from 30,000 to 50,000 cells per view field were estimated in Zone I biofilm micrographs; 1000 to 30,000 cells were estimated in Zone II biofilm micrographs, and up to 1000 cells were estimated in Zone III biofilm micrographs. Similarly, the numbers of the dead bacteria were estimated from the red fluorescence. The fraction of the dead cells in a given zone was calculated as

(2) $$\begin{array}{c} \%N_{d}=\frac{N_{d}}{N_{d}+N_{l}}\times 100,\;\left(2\right) \end{array}$$

where *N*_d_ is the number of dead bacteria, *N*_l_ is the number of live bacteria. The calculated fraction of dead bacteria in the negative control (PBS) was subtracted from the fraction of dead bacteria in the biofilm samples treated with different biocides.

### Planktonic Biocide Treatment

Planktonic *E. coli* cells that were used for experiments with biocides were first incubated overnight in LB medium. Two percent (v/v) of the overnight culture was transferred to a fresh rich growth medium supplemented with glucose (22.0 g of glucose, 1.88 g KH_2_PO_4_, 2.6 g Na_2_HPO_4_, 10.0 g peptocomplex, and 5.0 g yeast extract solubilized in 1 L of distilled water) and incubated for 2.5 h to obtain cell density of approximately 10^7^/mL. Next, the bacterial suspensions were either diluted or concentrated 100-fold. To concentrate or dilute bacterial suspensions the cultures were centrifuged at 8000 × *g* for 5 min and pellets were re-suspended in appropriate lower or higher volume of PBS to obtain the final cell concentrations. Next, equal volumes of undiluted, diluted, and concentrated cultures were centrifuged at 8000 × *g* for 5 min and the pellets were re-suspended in 300 μL of antimicrobial agent or in 300 μL PBS for a negative control. Cell suspensions were treated for 20 min and then centrifuged at 8000 × *g* for 5 min. The pellet was re-suspended in 300 μL PBS and stained with LIVE/DEAD stain according to the manufacturer instructions. Ten microliters of stained cell suspension were transferred to microscopic glass slides and covered with cover glass (20 × 20 mm). The fraction of the dead cells in planktonic phase was calculated as described for cells in biofilms.

### Biofilm Viscoelastic Properties

To obtain enough material for rheological measurements 5 mL of overnight bacterial culture was evenly spread over agar solidified rich growth medium supplemented with glucose or lactose (1.88 g KH_2_PO_4_, 2.6 g Na_2_HPO_4_, 10 g peptocomplex, 5.0 g yeast extract, 20 g agar, and 22.0 g glucose or 23.0 g lactose monohydrate solubilized in 1 L of distilled water) in a glass petri dish (diameter 23 cm) and incubated at 37°C for 24 h. Dynamic rheological measurements were performed on a rotational rheometer Physica MCR 302 (Anton Paar, Graz, Austria) at (20.00 ± 0.01)°C. The rheometer was equipped with the plate–plate measuring system (diameter 49.975 mm). Approximately 0.7 mL of biofilm material was applied to the measuring system. Oscillatory amplitude sweep measurements were conducted at the angular frequency ω of 10^-1^ s and the strain γ ranging from 0.001 to 1000% in 20 logarithmically spaced steps. Viscosity curves were measured at shear rates ranging from 0.01 to 1000^-1^ s in 40 logarithmically spaced steps with a time delay of 10 s between the successive measurements (Stojkovic et al., 2015). All rheological experiments were done in triplicates.

### Statistics

The average values and standard errors were calculated. In experiments where the effectiveness of biocides was tested three independent biological experiments each made in triplicate were evaluated. For statistical analysis two-tailed *t*-test assuming equal variances were used.

## Results

### Biofilm Surface Coverage

**FIGURE 1:**
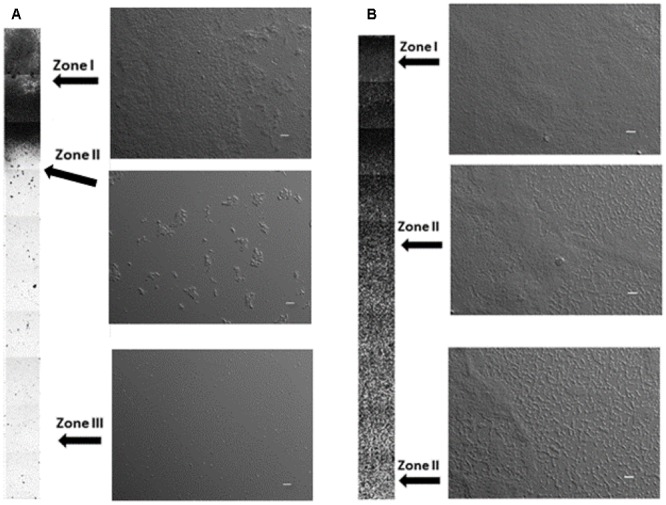
Biofilms grown on glass surface in the rich medium supplemented with glucose **(A)**, biofilms grown on PMMA surface in the rich medium supplemented with lactose **(B)** after 48 h of incubation. Zone I – arbitrarily designed high-density region where biofilm covered 90% or more of the available surface, Zone II – biofilm covered between 10 and 90% of the available surface, Zone III – biofilm surface coverage was less than 10%. Columns represent a low magnification DIC micrographs taken every 0.8 mm in the vertical direction from the water–air interphase (total depth 20 mm). Three representative higher magnification micrographs for different zones are shown on the right of the columns. Scale bar on micrographs represents 20 μm.

Two different biofilm systems were tested in this study: glucose/glass and lactose/PMMA. The *E. coli* biofilm grown on glass surface in the rich growth medium supplemented with glucose is shown in **Figure 1A**. The fraction of surface covered with biofilm decreased from the water–air interface to the bottom of the test tube producing a gradient in surface coverage in the vertical direction. The surface covered with biofilm was arbitrarily divided into three regions of high, medium, and low surface coverage. In Zone I biofilms formed a rather uniform high-density structures up to three layers thick which on average covered 95% of the available surface. Cells were embedded in the extracellular matrix structure. The length of Zone I increased after 72 h of incubation. There was a rather step gradient in surface coverage from Zone I to Zone III biofilm. In Zone II aggregates of attached bacteria were separated by individual cells attached to the glass surface. Cells in the aggregates were partially embedded in the extracellular matrix. In Zone III few microaggregates were present; mostly individual cells were attached to the surface that were not covered with extracellular matrix.

The biofilms grown on rich medium supplemented with lactose on PMMA were very different (**Figure 1B**). Bacteria formed well-developed dense biofilms only after 48 h of incubation. The length of the Zone I was significantly larger. Biofilms formed up to five layers thick continuous biofilm structures with smooth surface. The individual cells were embedded in the matrix. In Zone II bacterial cells produced aggregates of attached cells that form an interconnected biofilm network. Larger aggregates, two to three layers thick, were interspersed in the interconnected network and were covered with extracellular matrix. The number of large aggregates decreased in the vertical direction. Only few separated individual cells were observed in Zone II. The average surface density in the Zone II was higher compared to the respective zone of the glass surface. There was no Zone III biofilms after 48 h of incubation. The growth dynamics of the two biofilms is given in **Table 1**. The total area covered by the two biofilms increased during the incubation. When biofilms grew on PMMA surface in the rich medium supplemented with lactose significantly more biofilm structures formed compared to biofilms grown on glass surface supplemented with glucose. For instance, after 72 h of incubation the total surface covered with lactose grown biofilm structures on PMMA was 453.4 mm^2^ compared to 86.9 mm^2^ on glucose grown biofilm on glass surface.

**Table 1:** Surface area covered with biofilm after 24, 48, and 72 h of incubation of *E. coli* in rich growth media supplemented with glucose on glass surface or lactose on PMMA surfaces.

Biofilm covered surface (mm^2^)								
Medium and material	Rich medium with glucose, glass				Rich medium with lactose, PMMA			
Time (hours)	Zone I	Zone II	Zone III	Total surface	Zone I	Zone II	Zone III	Total surfaces
24	4.09 ± 0.38	5.98 ± 0.37	2.14 ± 0.15	12.21	0	1.29 ± 0.32	6.49 ± 0.99	7.78
48	34.86 ± 2.04	11.6 ± 1.56	2.60 ± 0.28	49.06	73.71 ± 5.02	192.96 ± 10.15	0	266.67
72	36.19 ± 1.55	45.43 ± 5.09	5.33 ± 0.73	86.95	231.85 ± 1.47	221.55 ± 5.08	0	453.40

*The average values and standard errors are given (*n* = 9).*

### Biocide Effectiveness Anti-correlates with Biofilm Surface Coverage

**FIGURE 2:**
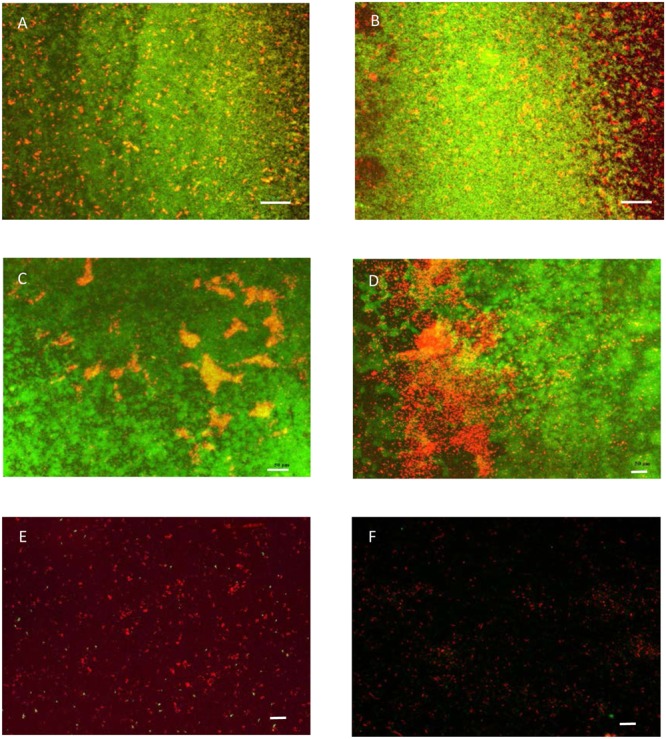
The live/dead assay on cells in Zone I **(A,B)**, Zone II **(C,D)**, and Zone III **(E,F)** biofilms. Biofilms were grown for 48 h in rich growth medium supplemented with glucose on glass surfaces and treated with Klercide B for 20 min. Scale bars in **(A)** and **(B)** represent 100 μm, in other panels they represent 50 μm.

**FIGURE 3:**
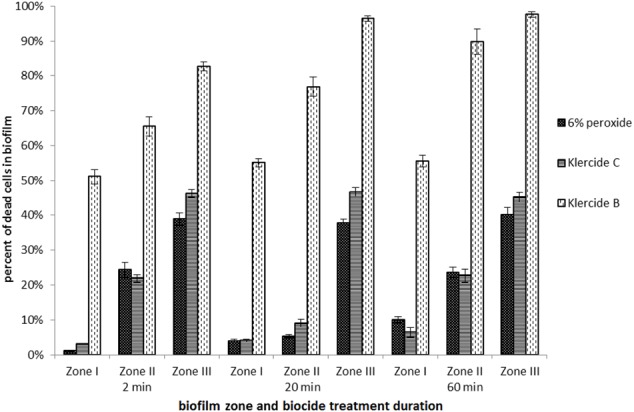
Fraction of the dead cells in different biofilm zones. Biofilms of *E. coli* were grown in the rich medium with glucose on glass surface for 48 h. Different biocides were added to for 2, 20, and 60 min. The average values and standard errors are given (*n* = 9).

The results of live/dead assay on glucose grown biofilms on glass surface treated for 20 min with Klercide B are given in **Figure 2**. The red cells represent cells with compromised membrane after treatment with Klercide B. The fraction of the dead cells was highest in Zone III biofilms and decreased toward Zone I biofilms. Although most of the cells in Zone III were killed, there was nevertheless a significant number of green cells present after treatment with Klercide B. The results of the biocide treatments suggested that higher surface coverage anti-correlated with biocide effectiveness. To check this further various biocides and treatment durations were tested (**Figure 3**). The effectiveness of all biocides was inversely proportional to the biofilm surface coverage; it was low in Zone I and increased in Zones II and III. The most effective treatment was with Klercide B. The treatments with Klercide C or H_2_O_2_ gave comparable results. On average Klercide C slightly outperformed lab-made H_2_O_2_ biocide. The increased time of Klercide B treatment improved biocide effectiveness. However, the results for Klercide C and H_2_O_2_ were less clear. For example, in Zone I the duration of treatment with Klercide C and H_2_O_2_ increased the effectiveness of biocide; however, there was no significant effect in Zone III with prolonged duration of the treatment.

**FIGURE 4:**
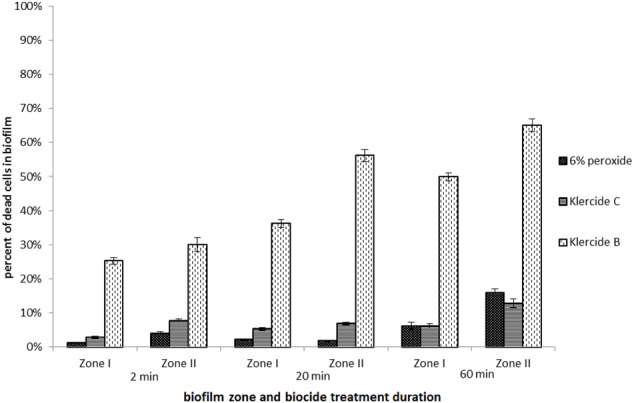
Effectiveness of different biocides. Biofilms of *E. coli* grown in the rich medium supplemented with lactose on PMMA surface for 48 h. Biocides were added for 2, 20, and 60 min. The average values and standard errors are given (*n* = 9).

Overall the effectiveness of different biocides was much lower when biofilms were grown in the rich medium with lactose on PMMA (**Figure 4**). For all tested biocides the effectiveness was higher after 60 min of treatment as compared to 2 min of treatment. For example, in the case of Klercide B the effectiveness in Zone I increased twofold from 26% dead cells after 2 min of treatment to 50% after 60 min, which is still considered low for an effective biocide treatment. The effectiveness of biocides in lactose/PMMA biofilms was lower compared to glucose/glass biofilms. In both biofilm systems (glucose/glass and lactose/PMMA) the most effective treatment was with Klercide B.

### Biocide Effectiveness in Planktonic Suspensions

**FIGURE 5:**
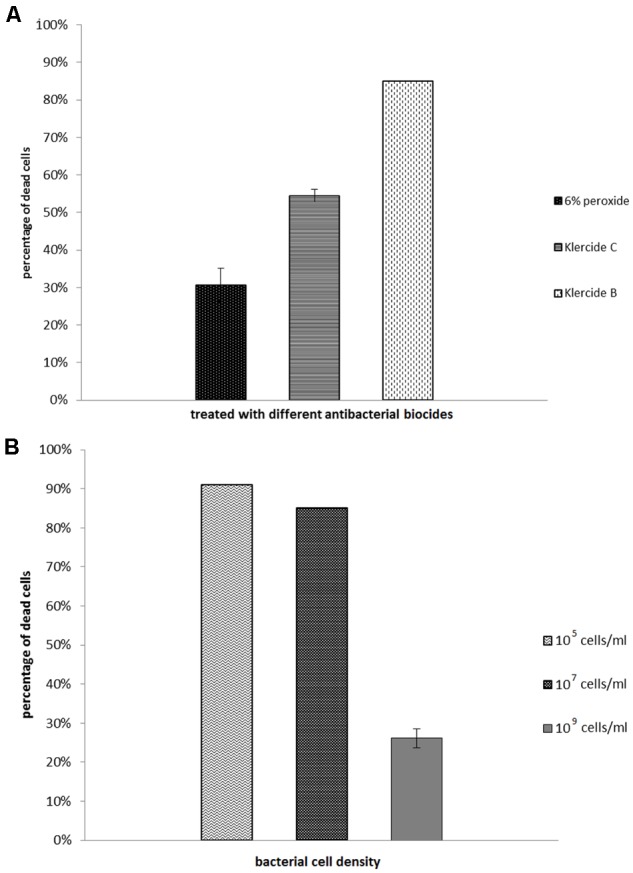
Fractions of the dead cells in planktonic culture of *E. coli* grown in the rich medium with glucose. Cells were grown to cell density of 10^7^ cells/mL and treated with different biocides **(A)**. Cells treated with Klercide B at different cell densities **(B)**. Bacterial suspensions with different cell density were treated with biocides for 20 min. The average values and standard errors are given (*n* = 9).

The effectiveness of different biocides tested on planktonic exponentially grown *E. coli* suspensions are given in **Figure 5A**. The most effective was Klercide B. The effectiveness of biocides was larger in dilute planktonic cell suspensions (**Figure 5B**). The effectiveness at low cell densities (e.g., 10^5^ cells/mL) was comparable to biocide effectiveness in Zone III biofilms. If the density of the planktonic culture increased the effectiveness of the biocide decreased significantly and when the cell density was 10^9^ cells/mL it was comparable to the biocide effectiveness in high-density Zone I biofilms.

### Viscoelasticity of *E. coli* Biofilms

**FIGURE 6:**
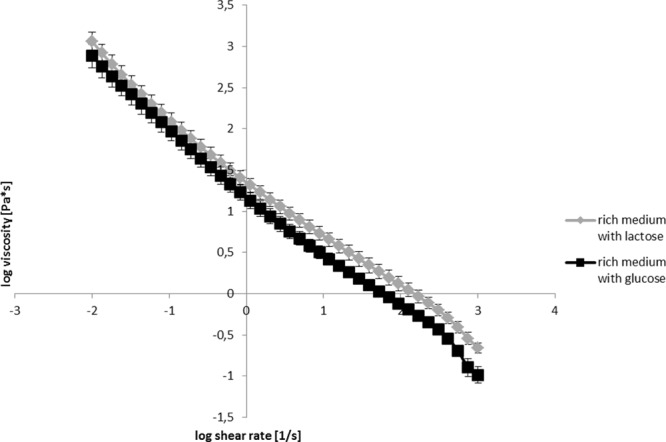
Viscosity curves for the confluent biofilm of *E. coli* grown in the rich medium with glucose or lactose after 24 h of incubation on agar plates.

Although surface coverage in Zone I biofilms were approximately equal in glucose/glass and lactose/PMMA biofilms (95.3 and 93.8%, respectively) and the thicknesses of the two biofilms were comparable, the effectiveness of the biocide treatments were lower in lactose/PMMA biofilms. In addition it took longer to obtain approximately the same killing effectiveness with Klercide B in lactose/PMMA biofilms in Zone I biofilms. This could be due to different diffusion rates of biocides in the two biofilms. According to Stokes–Einstein diffusion equation *D =* (*k*_B_*T*)/(*6*πη*a*), where *D* the is diffusion constant, *k*_B_ is the Boltzmann constant, *T* is the temperature, η is the viscosity, and *a* the radius of a particle, slower diffusion at a given temperature and size of the diffusing molecule is a direct consequence of a more viscous environment. To check for this the viscosities of the two confluent biofilms grown on agar surface were measured. The viscosity curves of the *E. coli* biofilms grown on glucose or lactose rich medium are given in **Figure 6**. The viscosity curve for the biofilm grown on lactose was consistently higher at all shear rates tested (over five orders of magnitude). The viscosity curves of both biofilms indicate a strong pseudoplastic behavior typical of a biofilm behavior.

**FIGURE 7:**
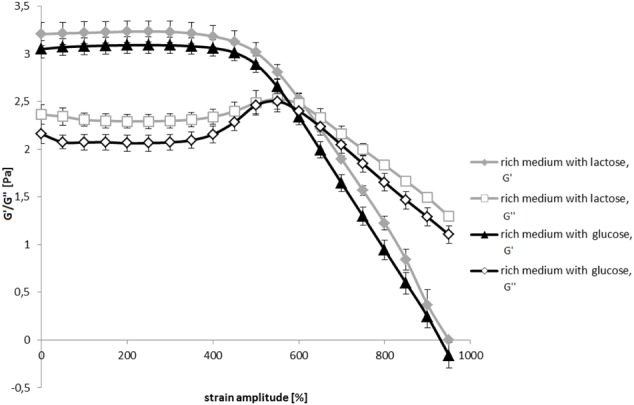
Viscoelastic moduli G′ and G″ as a function of strain amplitude for the confluent *E. coli* biofilms grown in the rich medium supplemented with glucose or lactose after 24 h of incubation on agar plates.

The two biofilms had different consistencies. The storage and loss moduli of biofilms grown in the rich medium with glucose or lactose are given in **Figure 7**. The behavior of the two biofilms is typical for viscoelastic gel materials. Both the storage modulus (*G*′) and the loss modulus (*G*″) were higher in lactose biofilms compared to glucose biofilms. From oscillatory tests one can notice that the structure of the biofilm grown on glucose is more fragile and starts to break at lower shear stress than the biofilm grown on lactose. Also the flow point, when *G*′ and *G*″ curves cross, is reached at a lower shear strain in glucose biofilms. This suggests that gel structure of the biofilm grown on lactose is stronger and more viscous which could explain lower effectiveness of biocides compared to glucose biofilms.

## Discussion

In this work the biocide effectiveness was correlated to the surface area covered by *E. coli* biofilms. In spite of extensive research of inadequate biocide effectiveness in biofilms, there are no systematic studies correlating biofilms surface area to biocide effectiveness. The obtained results imply a strong anti-correlation between the effectiveness of the biocide and the fraction of surface covered with the biofilm. Higher the fraction of the surface covered with the biofilm, lower the effect of the biocide. This was observed for all biocides tested.

Although there was a clear anti-correlation of biocide effectiveness with biofilm surface coverage this alone cannot explain the results obtained. The different biocide effectiveness in the three zones is further modified by different concentrations of the extracellular matrix present. Cells in Zone I were covered with extracellular matrix, which increases their resistance to the oxidative biocides. On the other hand, in Zone III individual cells were not embedded in the extracellular matrix. These cells were most susceptible for the action of biocides. It is, however, questionable if cells attached to the surface in Zone III can be considered biofilm structures. Although cell attachment is a necessary condition it is not a sufficient condition for mature biofilm formation. The results suggest that biofilm biocide resistance is an acquired property that increases with biofilm maturation. The antibacterial effect in Zone III was comparable to the effect of the biocide in low density *E. coli* planktonic cultures. In contrast in high-density planktonic cultures the effectiveness of biocides was similar to high-density Zone I biofilms. Similar observations have been made by Kirby et al. (2012) who showed that high-density planktonic growth stimulates the same level of resistance to antimicrobial agents as adherent biofilms. The effectiveness of biocides was time dependent and increased with the duration of the biocide treatment (**Figures 3, 4**).

Structural properties of biofilms are to a large extent determined by the environment in which biofilms grow. For example, changing organic carbon composition in the growth medium of *Bacillus subtilis* had a dramatic impact on the extracellular matrix production and composition (Dogsa et al., 2013). Different extracellular matrix composition will in turn affect viscosity of the extracellular matrix and consequently biocide effectiveness (Rühs et al., 2013). It has been suggested that viscous environment can induce tolerance to antibiotics within planktonic bacterial populations to the levels found in biofilms. For example, *Pseudomonas aeruginosa* and *Staphylococcus epidermidis* exhibited enhanced tolerance to biocides when grown in 30% poloxamer gel (Gilbert et al., 1998; Wirtanen et al., 1998). Similarly, increased tolerance of *Pseudomonas* and *Candida* to antimicrobials in viscous media supplemented with poly(vinylpirrolidone) (PVP) was observed (Chan, 1998; Kostenko et al., 2007). The results of the biofilm viscoelastic measurements of biofilms grown on glucose or lactose on agar surface (**Figures 6, 7**) suggest the role of carbon source in structuring the extracellular matrix. The lactose grown biofilms which produced stronger gels and had higher viscosities may be less susceptible for biocide tratment.

All tested biocides contained oxidants (chlorine dioxide or H_2_O_2_). The presence of biofilm extracellular matrix material will reduce the effectiveness of biocides that rely solely on oxidative stress such as Klercide C or 6% H_2_O_2_ solutions (Finnegan et al., 2010). It is expected that in biofilms with larger surface coverage (e.g., Zone I), where more extracellular matrix is produced, this will be more pronounced. Consistently, the effectiveness of Klercide C and 6% H_2_O_2_ was lowest in high-density biofilms. The most effective antibacterial agent was Klercide B which combined the oxidative damage induced by chlorine dioxide with surface activity of CTAB. This was particularly noticeable in biofilms with large surface area. For example, in Zone I Klercide B was 22-fold more effective than Klercide C, it was 4.5-fold more effective in Zone II, whereas in Zone III it was only twofold more effective after 2 min of treatment. This further emphasizes a differential effectiveness of biocides in small and large aggregates of biofilms.

## Conclusion

The results clearly indicate that oxidative biocides work efficiently on a single attached cell or small aggregate of attached cells, but significantly less on biofilms that cover large surface areas. Biofilm biocide resistance increases with maturation which correlates with extracellular matrix production. Thus, more dense attached structures present lower log reductions (i.e., lower percentages of dead cells) compared to less dense ones. The observations further suggest that changing viscoelastic properties of biofilms may allow for a better diffusion and therefore increased effectiveness of biocide treatment, a hypothesis worth further testing. For applications of biocides in biofilm control it might therefore be beneficial to reduce the density of the biofilm prior to the biocide application. This could be achieved for example by mechanical scraping, using scrub brushes, increased flow shear stress, or laser activated irrigation, which will reduce the size and cohesiveness of the biofilm aggregate prior to the application of the biocide. In reporting the efficiency of the biocide treatments the density of the biofilm (e.g., fraction of the surface covered by biofilms and/or cellular concentration) should be reported to ensure better reproducibility of the data in different laboratories.

## Author Contributions

SB performed experiments, analysis, conducted the work, and was involved in designing of the work, writing, and interpretation of the data. MK was involved in designing of the work, interpretation of the data, and writing. DS was involved in designing of the work, interpretation of the data, writing, submission of the manuscript, and ensured that questions related to the accuracy or integrity of any part of the work are appropriately investigated and resolved.

## Conflict of Interest Statement

The authors declare that the research was conducted in the absence of any commercial or financial relationships that could be construed as a potential conflict of interest.
